# Supplementary material for: RGS5 Attenuates Baseline Activity of ERK1/2 and Promotes Growth Arrest of Vascular Smooth Muscle Cells
Source: Cells. 2021 Jul 11;10(7):1748. doi: 10.3390/cells10071748 (PMC8306326; doi:10.3390/cells10071748)
Supplement: Supplementary file 1 [file cells-10-01748-s001.zip › cells-1258317-supplementary.pdf]

Supplement 1

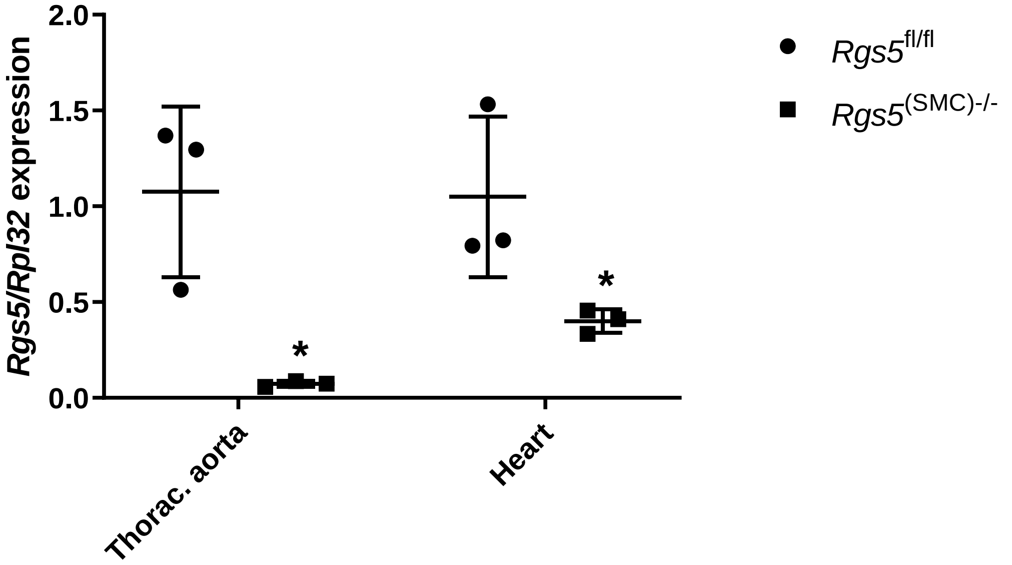

Figure S1: Verification of the *Rgs5* knockout in mouse tissues

8-10 weeks old *Rgs5*$^{fl/fl}$ male SM-MHC-CreER^{T2} -*Rgs5*$^{fl/fl}$ mice were injected with either Miglyol (*Rgs5*$^{fl/fl}$) as control or Tamoxifen (*Rgs5*$^{(SMC)-/-}$) to induce genetic ablation of *Rgs5*. 3 weeks later, the *Rgs5* expression in the thoracic aorta and heart tissue was analyzed by qPCR. *Rpl32* was used as a reference gene (*p<0.05, n=3).

Supplement 2

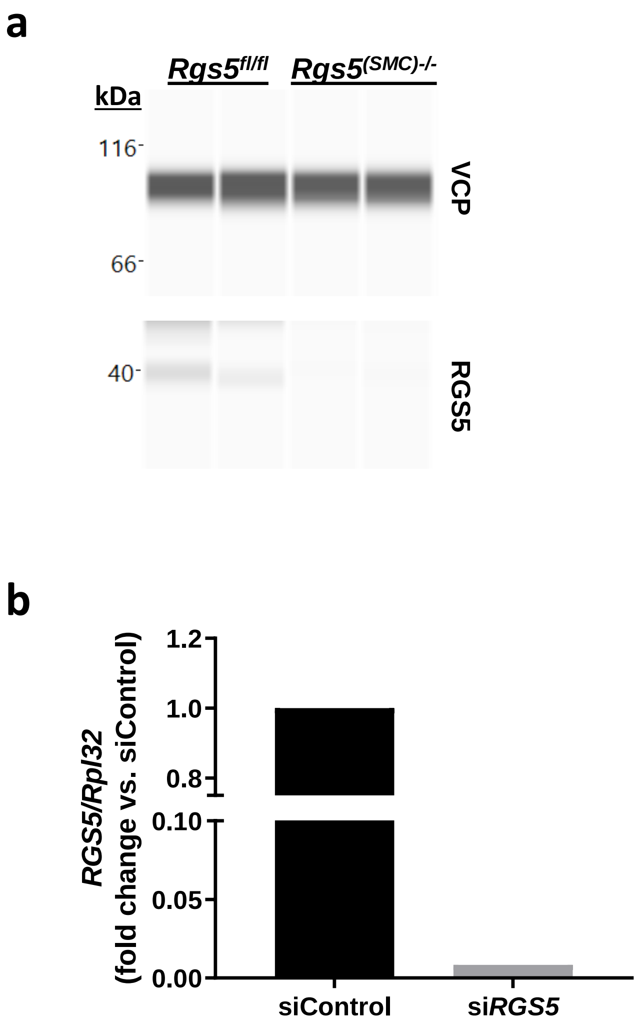

Figure S2: Knockdown of *Rgs5* in VSMCs

(a) RGS5 was detected in lysates of caudal arteries isolated from *RGS5*$^{fl/fl}$ and *RGS5*$^{(SMC)-/-}$ after separating the proteins by capillary electrophoresis to verify the specificity of the anti-RGS5 antibody. Under these conditions, the antibody detects an antigen with an estimated molecular weight of ~40kDa, which was barely detectable in arteries from *Rgs5*$^{(SMC)-/-}$ mice. Valosin containing protein (VCP) served as reference. **(b)** HUASMCs were transfected with either control siRNA (siControl) or *RGS5*-targeting siRNA (si*RGS5*). qPCR analysis was performed 72 h after transfection to verify knockdown efficiency. *Rpl32* was used as a reference.

Supplement 3

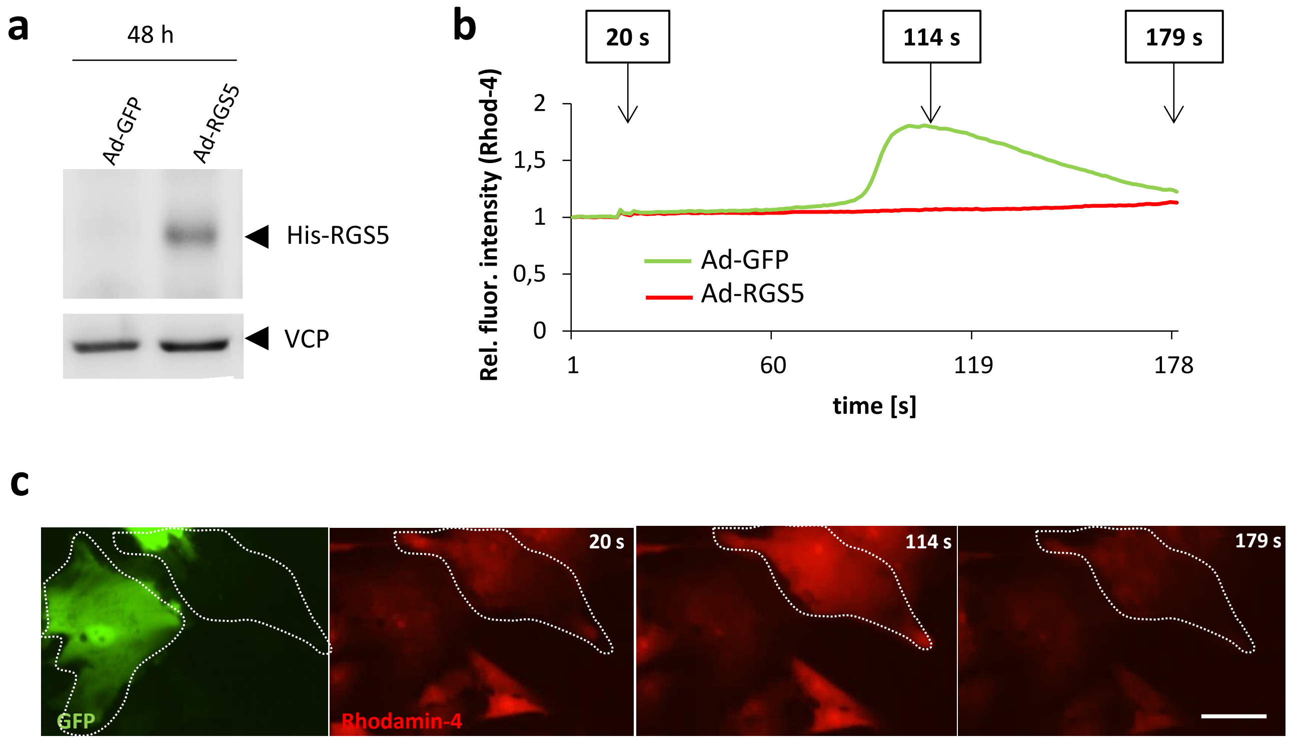

Figure S3: Over-expression of *RGS5* in HUASMCs

HUASMC were transduced with adenoviruses for overexpression of GFP (Ad-GFP) or His-tagged RGS5 and GFP (Ad-RGS5). **(a)** His-RGS5 was detected by Western blot-based analyses utilizing an anti-His antibody. VCP was used as a loading control. **(b)** Intracellular calcium release of HUASMCs was induced by 10 nM bradykinin, detected by Rhodamine-4 (red fluorescence) and recorded by time-lapse fluorescence microscopy. The green curve represents the response of HUASMCs transduced with Ad-GFP, while the red curve shows the calcium release in HUASMCs overexpressing RGS5. **(c)** Representative images of Ad-RGS5-transduced HUASMCs compare the change in Rhodamin-4-based fluorescence of two adjacent cells (dotted lines). The leftmost cell shows robust GFP fluorescence (indicating successful transduction with Ad-RGS5) and no response to bradykinin (red fluorescence) while GFP was not detected in the second cell, which shows an increase in the calcium/Rhodamin-4 fluorescence intensity (scale bar: 50 µm).

Supplement 4

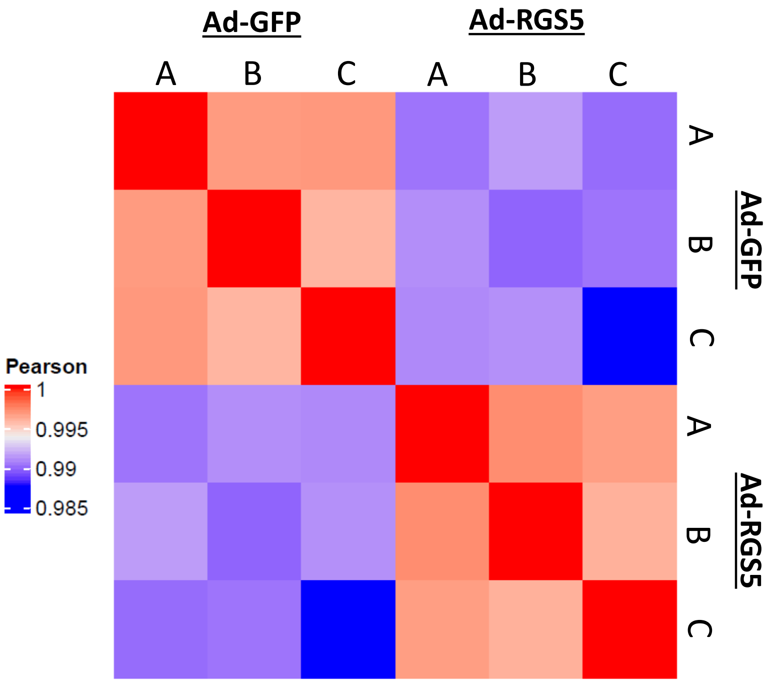

Figure S4 – Principal component analysis of microarray results comparing HUASMCs overexpressing *RGS5* and *GFP*

HUASMCs were transduced with adenoviruses to overexpress *GFP* or *RGS5*, respectively. Three RNA samples of each group were compared. The presented heat map indicates an adequate overall match of samples within a given experimental group.

Supplement 5

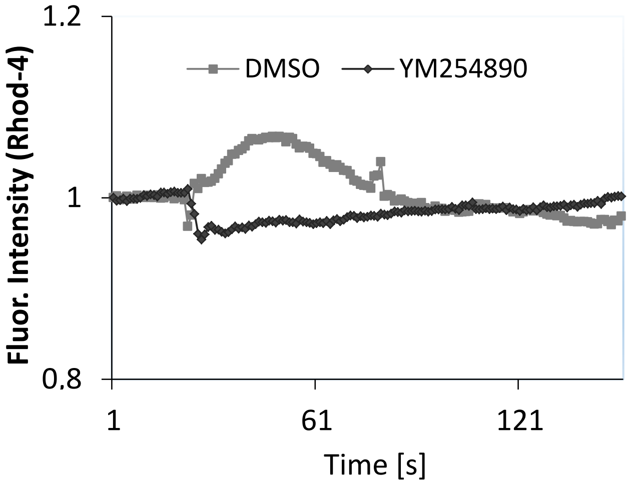

Figure S5: G$\alpha_{q/11}$ inhibitor (YM254890) blocks AngII-provoked calcium release in HUASMC.

Angiotensin II-induced (1 µM) calcium release was detected by Rhodamine-4 fluorescence in HUASMCs treated with G$\alpha_{q/11}$ inhibitor YM-254890 (2 µM).

Supplement 6

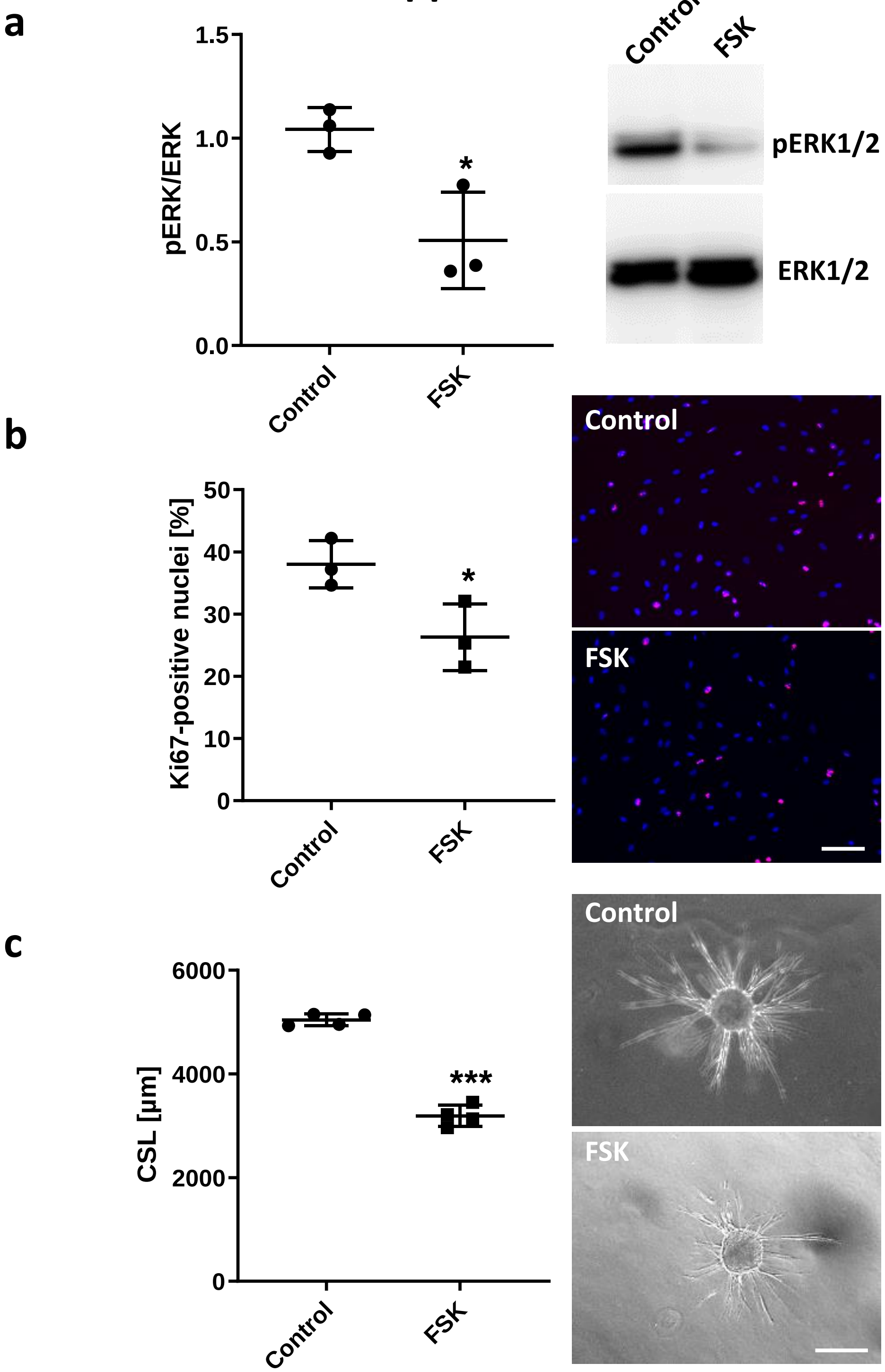

Figure S6: Effect of Forskolin on ERK phosphorylation, proliferation and migration of HUASMCs

(a) Lysates of HUASMCs treated with 25 µM Forskolin (FSK) for 24h were analyzed by immunoblot-based techniques to detect ERK1/2 (loading control) and pERK1/2 (*p<0.05, n=3). **(b)** Proliferation of HUASMCs was determined by immunofluorescence-based detection of the proliferation marker Ki67 24h after treatment with FSK (*p ≤ 0.05, n = 3; Ki67: red, DAPI: blue, scale bar: 100 µm). **(c)** Outgrowth of 25 µM FSK-treated HUASMCs from collagen gel-embedded spheroids was determined by measuring the cumulative length of all “sprouts” originating from a spheroid (CSL, *p ≤ 0.05, n=4, 10 spheroids per group and experiment were measured, scale bar: 200 µm).

Supplement 7

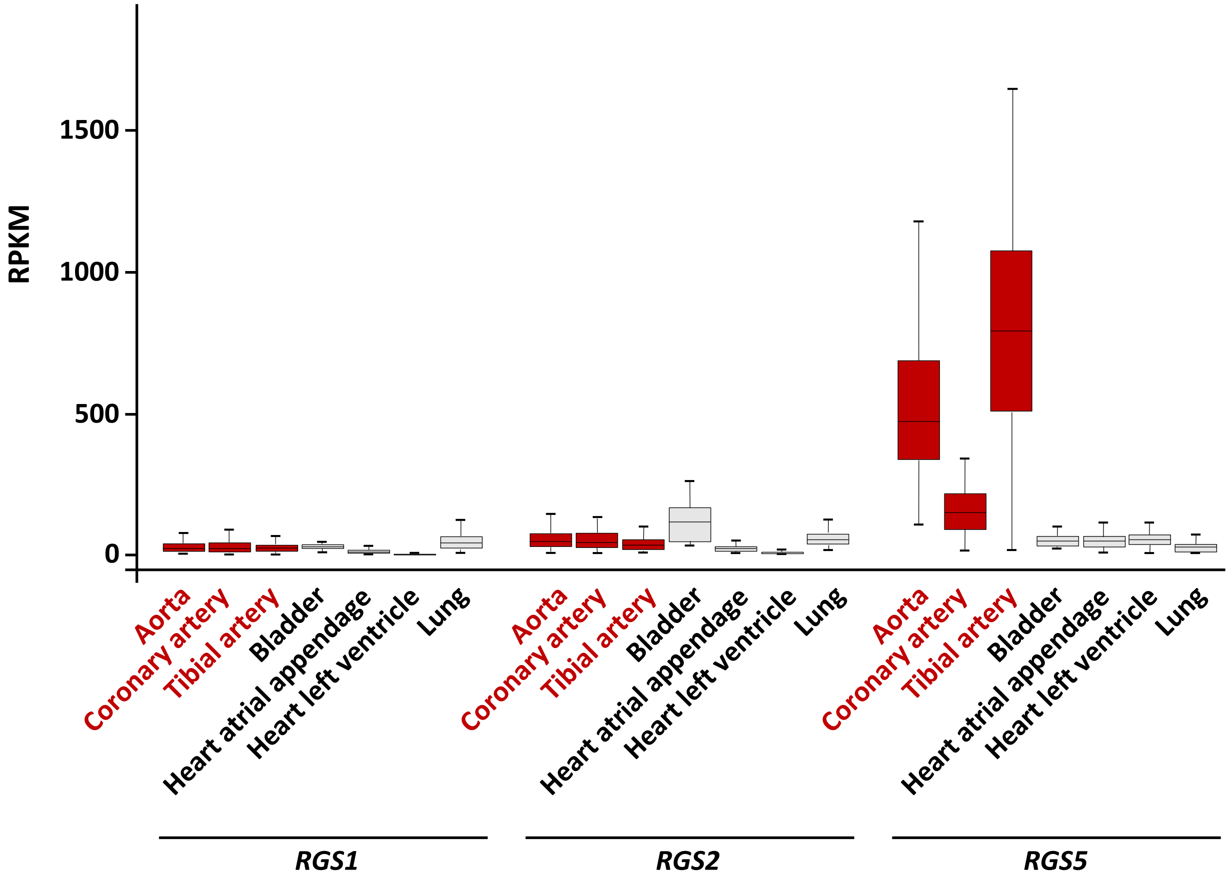

Figure S7: Comparison of the expression level of *RGS1, 2 and 5* in different human tissues

The data was extracted from the GTEx database as published (Science. 8 May 2015: Vol 348 no. 6235 pp 648-660. DOI: 10.1126/science.) and presents an analysis of RNA sequencing data from 1641 samples across 43 tissues and 175 individuals. The expression values are shown in RPKM (Reads Per Kilobase of transcript per Million mapped reads), calculated from a gene model with isoforms collapsed to a single gene. No other normalization steps have been applied. Box plots are shown as median and 25th and 75th percentiles.

Supplement 8

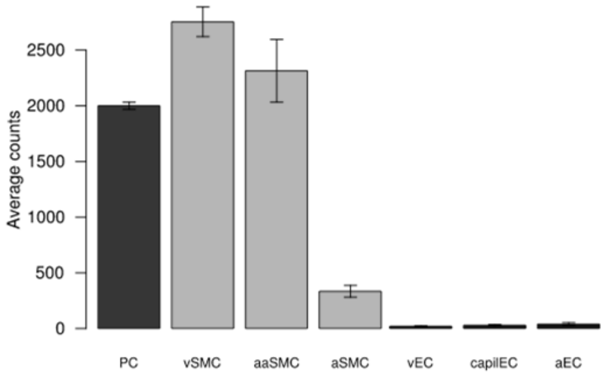

Figure S8: Comparison of the expression level of *RGS5* in individual vascular cell types of the mouse brain.

The data was gathered from the following database http://betsholtzlab.org/VascularSingleCells/database.html as published (Vanlandewijck, M., He, L. *et al*. *Nature*, 554, 475-480, 2018; He, L., Vanlandewijck, M. *et al*. Data Descriptor: *Scientific Data*, Volume 5, Article number: 180160, 2018) and compared the cell type-specific expresssion level of *Rgs5* in the mouse brain based on single-cell sequencing data (scRNAseq). Individual cell types were identified by a set of markers specific for the indicated cell types (PC – pericytes, vSMC – venous smooth muscle cells, aaSMC – arteriolar smooth muscle cells, aSMC – arterial smooth muscle cells, vEC – venous endothelial cells, capilEC – capillary endothelial cells, aEC – arterial endothelial cells).
